# Sensitivity and Predictive Value of 15 PubMed Search Strategies to Answer Clinical Questions Rated Against Full Systematic Reviews

**DOI:** 10.2196/jmir.2021

**Published:** 2012-06-12

**Authors:** Thomas Agoritsas, Arnaud Merglen, Delphine S Courvoisier, Christophe Combescure, Nicolas Garin, Arnaud Perrier, Thomas V Perneger

**Affiliations:** ^1^Division of Clinical EpidemiologyUniversity Hospitals of GenevaGenevaSwitzerland; ^2^Division of General Internal MedicineUniversity Hospitals of GenevaGenevaSwitzerland; ^3^Division of General PediatricsGeneva Faculty of MedicineUniversity Hospitals of GenevaGenevaSwitzerland; ^4^Department of PsychologyHarvard UniversityCambridge, MAUnited States

**Keywords:** Evidence-based medicine, information retrieval, medical literature, search strategy, PubMed, Medline, clinical queries, search filters, sensitivity, recall, positive predictive value, precision

## Abstract

**Background:**

Clinicians perform searches in PubMed daily, but retrieving relevant studies is challenging due to the rapid expansion of medical knowledge. Little is known about the performance of search strategies when they are applied to answer specific clinical questions.

**Objective:**

To compare the performance of 15 PubMed search strategies in retrieving relevant clinical trials on therapeutic interventions.

**Methods:**

We used Cochrane systematic reviews to identify relevant trials for 30 clinical questions. Search terms were extracted from the abstract using a predefined procedure based on the population, interventions, comparison, outcomes (PICO) framework and combined into queries. We tested 15 search strategies that varied in their query (PIC or PICO), use of PubMed’s Clinical Queries therapeutic filters (broad or narrow), search limits, and PubMed links to related articles. We assessed sensitivity (recall) and positive predictive value (precision) of each strategy on the first 2 PubMed pages (40 articles) and on the complete search output.

**Results:**

The performance of the search strategies varied widely according to the clinical question. Unfiltered searches and those using the broad filter of Clinical Queries produced large outputs and retrieved few relevant articles within the first 2 pages, resulting in a median sensitivity of only 10%–25%. In contrast, all searches using the narrow filter performed significantly better, with a median sensitivity of about 50% (all *P *< .001 compared with unfiltered queries) and positive predictive values of 20%–30% (*P *< .001 compared with unfiltered queries). This benefit was consistent for most clinical questions. Searches based on related articles retrieved about a third of the relevant studies.

**Conclusions:**

The Clinical Queries narrow filter, along with well-formulated queries based on the PICO framework, provided the greatest aid in retrieving relevant clinical trials within the 2 first PubMed pages. These results can help clinicians apply effective strategies to answer their questions at the point of care.

## Introduction

Searching the literature for evidence has become a central skill in clinical practice [1]. Physicians’ information needs are considerable [2-4], and evidence-based decisions often require the identification and appraisal of current research findings [5]. As Glasziou et al commented, “the use of search engines is now as essential as the stethoscope” [6]. However, retrieving relevant information has also become increasingly challenging, given the rapid expansion of medical knowledge: PubMed now comprises more than 20 million citations [7], and 2000–4000 more are added every day [6], including 75 clinical trials and 11 systematic reviews [8].

In the past decade, several solutions have been implemented to improve access to current research [9]. Preappraised resources, such as evidence-based medicine journals, or Web-based summaries such as UpToDate [10], have been adopted by many clinicians [11,12]. But these resources are limited by delayed processing [9,13], insufficient coverage [9,14], or cost [15,16]. Thus, PubMed remains the most popular search engine used to retrieve original studies [17-20], either alone or as a complement to preappraised resources [15,18].

**Figure 1:**
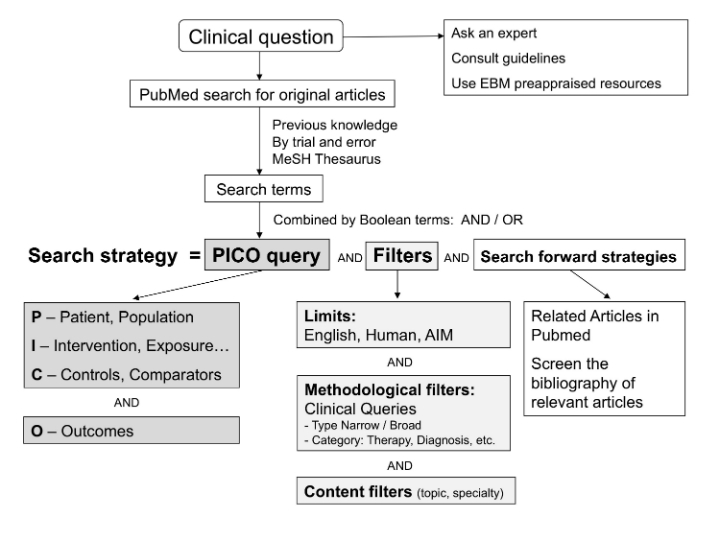
Framework for PubMed search strategies to answer clinical questions. AIM = Abridged Index Medicus (set of 119 core clinical journal titles [28]), EBM = evidence-based medicine, MeSH = Medical Subject Headings (a controlled vocabulary used for indexing articles in PubMed), PICO = population, interventions, comparison, outcomes.

However, searching PubMed is not as intuitive as searching other commonly used engines such as Google. While clinicians often perform short unstructured queries of 2–3 terms [21], these tend to produce large and diluted outputs. More efficient search strategies can be proposed (Figure 1), using existing search tools and based on expert recommendations. First, the clinical question is translated into search terms that are combined into a query using Boolean operators (eg, OR, AND) [22,23]. The use of the population, interventions, comparison, outcomes (PICO) framework helps formulate more precise queries that combine search terms for these four factors [22,24]. The size of the output can be further reduced using limits [25] or methodological filters, such as PubMed’s Clinical Queries [26], designed to retrieve high-quality randomized controlled trials for therapeutic interventions [27]. Finally, additional strategies, such as the use of the related articles link in PubMed, can identify studies based on a relevant article that was initially found (Figure 1 [28]).

Evidence regarding the performance of search strategies to answer clinical questions is scarce. A few small studies have assessed the impact of giving clinicians search tutorials on the retrieval of specific sets of articles identified by experts, but search strategies could not be compared, as clinicians were free to use their own informal strategies [29,30]. Other studies have reported on the performances of search filters in retrieving high-quality clinical trials, but these filters were assessed independently of user queries [31] or clinical questions [27]. To our knowledge, there is little evidence to help clinicians organize their searches and combine existing search tools into effective strategies that are applicable at the point of care.

In this study, we compared the performance of 15 PubMed search strategies in retrieving relevant clinical trials, as identified by high-quality systematic reviews on specific clinical questions. We devised these 15 search strategies by choosing and combining the following search components that are easily applicable at the point of care: formulation of queries using the PICO framework, use of Clinical Queries therapeutic filters (broad or narrow), use of several search limits, and use of PubMed links to related articles. Our aim was to identify search components and tools that would most likely help clinicians answer questions on therapeutic interventions at the point of care.

## Methods

### Sample of Systematic Reviews for the Identification of Relevant Articles

For a specific clinical question, a well-conducted systematic review is considered the gold standard for the identification of all relevant articles [31-33], since it implies a systematic and comprehensive search, as well as an appraisal of the articles’ scientific rigor. Moreover, recent systematic reviews address questions of clinical interest for which relative uncertainty exists and are thus a good proxy of the questions that may arise in real clinical practice.

We selected 30 clinical questions from a wide spectrum of topics of general interest, on the basis of 30 recent Cochrane systematic reviews (Table 1, Multimedia Appendix 1). We favored Cochrane reviews because of their well-established quality and methodological rigor [34-36], and focused on reviews that included randomized controlled trials, as these constitute the highest level of evidence for questions about therapeutic interventions. In February 2010, we searched the Cochrane Database of Systematic Reviews [37] for all reviews on therapeutic interventions published since the beginning of 2010. To focus on the most recent reviews, or reviews that were recently updated, we restricted the search to citations with the following record status: new review, new search, or conclusion changed. Of the 287 potentially eligible reviews, we excluded those with fewer than 4 studies (n = 113), those that were less relevant for a general audience (n = 82), and those with composite questions that would result in complex queries for clinical practice (n = 62). All clinical trials included in the remaining 30 reviews that were also retrievable in PubMed defined the subset of relevant studies used in our assessment.

### Extraction of Search Terms and Formulation of PICO Query

We selected search terms using a predefined procedure that we determined before applying the searches strategies. Following the PICO framework (Figure 1), we categorized the exact wording of the objective and selection criteria of each review’s abstract into four sections for the population, interventions, comparison, and outcomes [22,24]. When we could find no information in the abstract for one of these categories, we retrieved the relevant keywords from the methods section (eg, type of intervention or type of outcome). Then for each PICO category, we extracted the smallest set of search terms that best expressed the clinical question. This predefined procedure was performed by 2 assessors (TA and AM), both trained in clinical epidemiology and evidence-based medicine. In a learning phase, together they extracted search terms from a pilot sample of 10 systematic reviews. Then they independently extracted PICO search terms from the 30 reviews of the study sample and agreed on the smallest set of terms that best expressed the clinical questions. They resolved discrepancies by consensus. Finally, all coauthors, including experienced clinicians, approved the retained search terms. For half of the reviews, PICO’s comparison component shared common terms with interventions (eg, composite vs single intervention), and in these cases no term was retained for comparison. Examples of search term extraction are shown in Multimedia Appendix 2.

To obtain the final search query (Figure 1), search terms were combined with the Boolean operator OR within each PICO category, so as to increase sensitivity. Then all categories were combined with AND to retrieve only the citations matching all PICO elements [22-24]. For example, for a review on the effects of oral mucolytic agents in adults with stable chronic bronchitis or chronic obstructive pulmonary disease (COPD) [38], the final full PICO query was *((chronic bronchitis) OR COPD) AND mucolytics AND placebo AND exacerbations)*. Search terms consisting of groups of words (eg, chronic bronchitis) were put into parentheses, not in quotes. The detailed wording of all 30 queries can be found in Multimedia Appendix 3.

### Design of Search Strategies

The purpose and context of the search determine the choice of search strategy. Our objective was to identify search components and tools that could help clinicians build more effective strategies to answer questions at the point of care. Therefore, selecting sophisticated strategies used for performing systematic reviews [39], such as the Cochrane Highly Sensitive Search Strategy [40], would have been inappropriate. Since there is very little evidence on strategies adapted to our purpose, we devised our 15 search strategies (Table 2, Figure 1) based on the few expert recommendations available [22-24,26,27,29-31]. We favored search components and search tools that are applicable and easy to use in clinical practice.

We thus obtained strategies by varying the following parameters. First, the search query was either the full PICO query or a truncated PIC query that did not include terms of outcomes. Although the full PICO is usually recommended for clinicians to help them formulate more precise searches [22-24], such strategies may also miss relevant articles [40], as the outcomes are less often mentioned in the abstract or assigned Medical Subject Headings (MeSH) in PubMed. Moreover, formulating shorter PIC queries may be less burdensome in practice and may be more appropriate for clinicians who are interested in many potential outcomes. We entered search terms using PubMed’s automatic term mapping, which systematically searches each term in the MeSH thesaurus, as well as the “[all fields]” tag (Multimedia Appendix 2). We did not test strategies using the restriction to MeSH terms, as few clinicians manage to perform the necessary steps for this restriction in practice [21], and this proportion hardly increases after medical residents take a search tutorial [29].

Second, we combined these queries with either the broad or narrow filter of the Clinical Queries for therapeutic interventions. We chose Clinical Queries over other filters meant to improve the retrieval of high-quality randomized controlled trials [39] because Clinical Queries were specifically designed to help clinicians answer their questions on therapeutic interventions and are implemented in PubMed [27]. To use these filters, clinicians simply need to enter their search terms on PubMed’s Clinical Queries page [26]. After taking a search tutorial, about 80% of clinicians adopted the use of Clinical Queries [29]. Third, we repeated these strategies adding search limits [25] that restricted the searches to citations in English and research on humans. PICO searches with human and English limits were repeated with a further restriction to a set of 119 core medical journals listed in the Abridged Index Medicus (AIM) [28]. We did not further add the limit “clinical trials [pt],” as it would have been redundant with the Clinical Queries filters.

Finally, the last 3 searches tested the PubMed link to related articles, a search tool that identifies content similarity in the title, abstract, and index terms [41]. To use this tool in practice, clinicians must identify a citation they consider to be potentially relevant in a first search output (ie, a citation that best corresponds to their PICO question) and click on its link to related articles. They can then scan a new search output with citations ranked by content similarity with the initial citation. To test this strategy, 2 assessors (TA and AM) screened the first page of search #11 (PICO, therapy narrow filter, limited to human studies in English), a search strategy that yielded short outputs (Table 2), and identified by consensus the 3 citations that were closest to the corresponding PICO question. They performed this selection being blinded to the citations included in the review, so as to correspond to the situation that clinicians encounter in practice. Search performance was assessed on each output of these 3 citations’ related articles. We performed this strategy based on 3 citations, instead of 1, to minimize the impact of the subjective component of the choice of initial citation.

### Analysis of Search Performance

**Figure 2:**
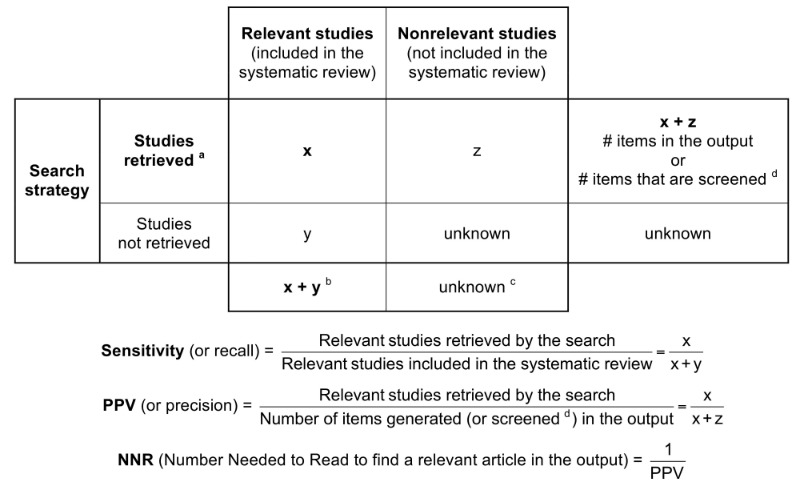
Definitions of the sensitivity and positive predictive value (PPV) of search strategies, when using a systematic review as the gold standard. ^a^ We included only the studies published before the date when the corresponding systematic review was assessed as up-to-date. ^b^ We excluded from this total the studies that are not indexed in PubMed, as they cannot be retrieved by any search strategy. ^c^ This total is unknown, because it is not limited to the studies that were explicitly excluded from the systematic review. ^d^ When only a limited number of items in the output are screened (eg, 40 items, or 2 pages of PubMed’s output), then practically this number becomes the true denominator of PPV.

We applied all 15 strategies for each of the 30 clinical questions (450 searches in total). We restricted each search to the date when the corresponding review was assessed as up-to-date, so that the time frame of the search was the same as its corresponding review. For each search we collected the number of articles in the output, the number of gold standard articles retrieved, and their position in the output. Then we computed the sensitivity (also called recall: the proportion of relevant papers that were retrieved) of each search and its positive predictive value ([PPV], also called precision: the proportion of the output corresponding to relevant papers), as defined in Figure 2 [27,31,33].

Because most physicians screen only 2 pages of the PubMed output, or at most 40 items [19,21], our primary outcomes for search performance were sensitivity and PPV for this cut-off. However, we also analyzed overall sensitivity and PPV on the full search output and further explored how these properties varied according to the cumulative number of items screened in the output.

We summarized the performance of the search strategies over the 30 clinical questions using nonparametric statistics and box plots. Filtered versus unfiltered searches were compared using the Wilcoxon signed rank tests (paired). Finally, we explored whether retrieval performances differed according to the characteristics of the corresponding review. All analyses were performed using R 2.12.1 software [42].

## Results

### Characteristics of Systematic Reviews and Corresponding PICO Queries

The 30 systematic reviews addressed a broad pattern of clinical topics and were produced by 15 different Cochrane groups, with a range of 1–4 reviews per group. Of these, 13 reviews (43%) had their record status labeled as new review, and 13 others (43%) as new search with no change to the conclusion and 4 (13%) with conclusion changed. They included a median of 15 studies (interquartile range [IQR] 7–23, range 5–49), of which 85% (IQR 66%–100%) were retrievable in PubMed (Table 1). This led to a median of 12 relevant clinical trials per review (IQR 7–18) that we considered to be the gold standard for each clinical question.

The predefined extraction procedure of search terms from the reviews’ abstracts led to a median of 2 terms (IQR 1–2) for population 1 term (IQR 1–2) for interventions, 1 term (IQR 0–1) for comparison, and 2.5 search terms (IQR 2–3) for outcomes. The required number of search terms was variable, as these terms were strictly tailored to the reviews’ questions. Indeed, these were sometimes more complex or consisted of grouping of words (eg, generalized onset tonic–clonic seizures). Overall, queries resulted in a median of 4 terms (IQR 3–6) for the PIC query and 7 terms (IQR 5–8) for the PICO query (for detailed wording, see Multimedia Appendix 3).

**Table 1:** Comparative performance assessed on the full search output (A) and on 2-page output (B) of 2 selected search strategies—one unfiltered (S1) and one using Clinical Queries narrow filter (S4)—to answer 30 clinical questions rated against 30 Cochrane systematic reviews.

Rev. No.	Review title	No. studies incl. in review	n (%) retrievable in PubMed	A. Search performance for the full output				B. Search performance for a 2-page output (maximum 40 items)			
				Sensitivity (%)		PPV^a ^(%)		Sensitivity (%)		PPV (%)	
				S1^b^	S4^c^	S1	S4	S1	S4	S1	S4
1	Carbamazepine versus phenytoin monotherapy for epilepsy	10	8 (80)	37.5	37.5	0.1	11.1	0.0	37.5	0.0	11.1
2	Chest physiotherapy for pneumonia in adults	6	5 (83)	100.0	80.0	1.9	12.5	0.0	80.0	0.0	12.5
3	Epinephrine injection versus epinephrine injection and a second endoscopic method in high risk bleeding ulcers	18	15 (83)	100.0	100.0	7.9	19.5	6.7	33.3	2.5	12.5
4	Extracranial-intracranial arterial bypass surgery for occlusive carotid artery disease	21	18 (86)	38.9	5.6	4.1	14.3	0.0	5.6	0.0	14.3
5	Fluticasone versus ‘extrafine’ HFA-beclomethasone dipropionate for chronic asthma in adults and children	9	8 (89)	100.0	100.0	18.6	42.1	100.0	100.0	20.0	42.1
6	Influenza vaccination for healthcare workers who work with the elderly	5	5 (100)	100.0	80.0	1.5	16.7	20.0	80.0	2.5	16.7
7	Mucolytic agents for chronic bronchitis or chronic obstructive pulmonary disease	29	18 (62)	83.3	66.7	11.5	18.8	22.2	38.9	10.0	17.5
8	Neuraminidase inhibitors for preventing and treating influenza in healthy adults	20	20 (100)	90.0	90.0	22.8	35.3	15.0	70.0	7.5	35.0
9	Adenoidectomy for otitis media in children	14	14 (100)	100.0	100.0	2.3	22.2	7.1	64.3	2.5	22.5
10	Antibiotics and antiseptics for venous leg ulcers	25	13 (52)	84.6	76.9	6.4	43.5	15.4	76.9	5.0	43.5
11	Antithyroid drug regimen for treating Graves’ hyperthyroidism	27	23 (85)	82.6	78.3	4.9	36.7	0.0	69.6	0.0	40.0
12	Artesunate versus quinine for treating severe malaria	6	4 (67)	100.0	100.0	8.3	33.3	50.0	100.0	5.0	33.3
13	Bed rest for acute uncomplicated myocardial infarction	17	11 (65)	54.5	27.3	19.4	60.0	54.5	27.3	19.4	60.0
14	Benzodiazepines for the relief of breathlessness in advanced malignant and non-malignant diseases in adults	7	4 (57)	75.0	50.0	10.7	40.0	75.0	50.0	10.7	40.0
15	Blood pressure lowering efficacy of beta-blockers as second-line therapy for primary hypertension	20	11 (55)	72.7	63.6	1.2	2.3	0.0	0.0	0.0	0.0
16	Blood pressure lowering efficacy of potassium-sparing diuretics for primary hypertension	8	7 (88)	100.0	71.4	1.2	2.5	0.0	0.0	0.0	0.0
17	Caffeine for asthma	7	7 (100)	85.7	71.4	66.7	83.3	85.7	71.4	66.7	83.3
18	Continuous subcutaneous insulin infusion (CSII) versus multiple insulin injections for type 1 diabetes mellitus	34	27 (79)	66.7	55.6	9.9	44.1	7.4	55.6	5.0	44.1
19	Effect of cyclosporine on blood pressure	17	17 (100)	47.1	47.1	9.3	11.9	17.6	23.5	7.5	10.0
20	Enteral versus parenteral nutrition for acute pancreatitis	8	8 (100)	100.0	100.0	3.9	30.8	12.5	100.0	2.5	30.8
21	Exercises for prevention of recurrences of low-back pain	13	13 (100)	84.6	84.6	0.5	2.7	0.0	0.0	0.0	0.0
22	Home-based versus centre-based cardiac rehabilitation	22	17 (77)	58.8	47.1	7.8	15.4	29.4	47.1	12.5	20.0
23	Immediate-release versus controlled-release carbamazepine in the treatment of epilepsy	10	10 (100)	80.0	80.0	11.8	34.8	30.0	80.0	7.5	34.8
24	Proton pump inhibitor treatment initiated prior to endoscopic diagnosis in upper gastrointestinal bleeding	6	3 (50)	33.3	33.3	0.9	14.3	0.0	33.3	0.0	14.3

25	Rectal 5-aminosalicylic acid for induction of remission in ulcerative colitis	38	33 (87)	90.9	84.8	10.3	32.6	12.1	39.4	10.0	32.5
26	Regular treatment with salmeterol for chronic asthma: serious adverse events	49	30 (61)	43.3	40.0	7.6	18.8	0.0	16.7	0.0	12.5
27	Rifabutin for treating pulmonary tuberculosis	5	4(80)	100.0	100.0	2.7	33.3	0.0	100.0	0.0	33.3
28	Serotonin receptor antagonists for highly emetogenic chemotherapy in adults	16	15(94)	46.7	46.7	2.2	7.3	6.7	13.3	2.5	5.0
29	Short-term treatment with proton pump inhibitors, H2-receptor antagonists and prokinetics for gastro-oesophageal reflux disease-like symptoms and endoscopy negative reflux disease	45	38 (80)	57.9	55.3	0.7	4.4	0.0	0.0	0.0	0.0
30	Therapeutic ultrasound for osteoarthritis of the knee or hip	5	5 (100)	100.0	100.0	9.4	29.4	100.0	100.0	12.5	29.4

^a ^Positive predictive value (ie, precision).

^b ^Unfiltered search No. 1 corresponding to a single population, interventions, comparison (PIC) query, without any filters or limits (see also Table 2).

^c ^Filtered search No. 4 corresponding to a PIC query, combined with the narrow therapy filter of PubMed’s Clinical Queries (see also Table 2).

### Performance of Search Strategies

We observed important differences in the sizes of the output across search strategies (Table 2). Unfiltered PIC queries resulted in the largest outputs, with a median of 173 items, while the PICO queries halved the output size. The use of the Clinical Queries broad filter reduced the output by about 20%, and use of the narrow filter reduced it by about 80%. In contrast, searches based on related articles typically retrieved hundreds of articles.

**Figure 3:**
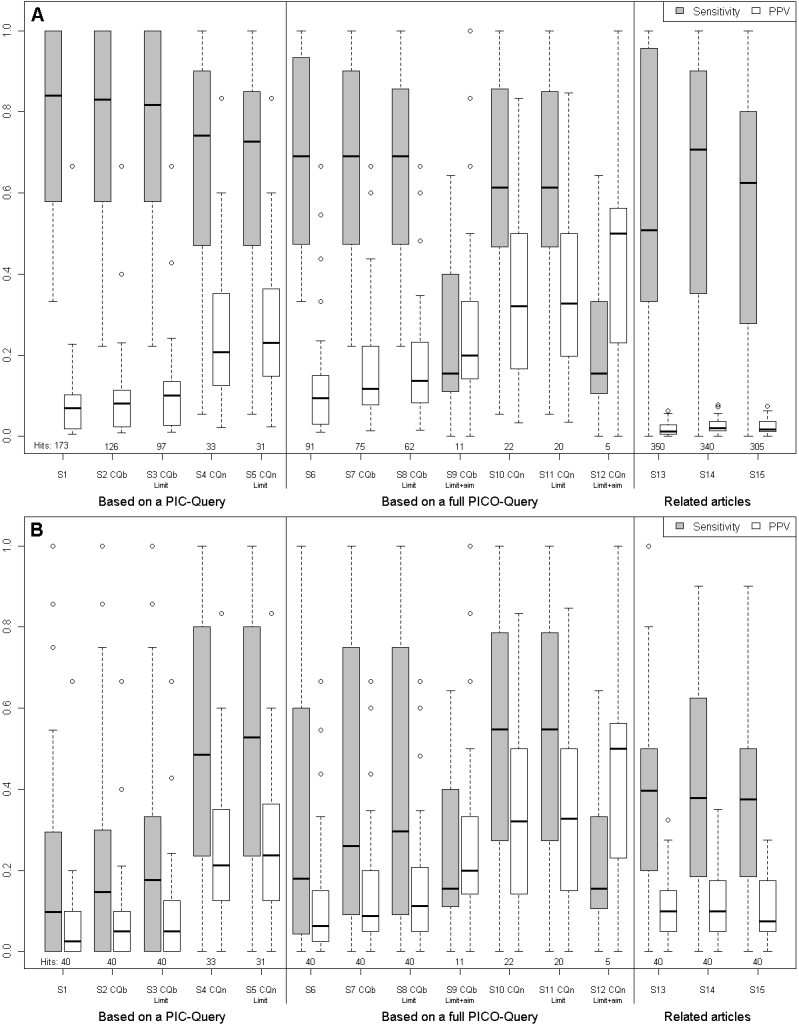
Compared sensitivity and positive predictive value (PPV) of the 15 search strategies (S) tested, for (A) the full search output and (B) the first 2 PubMed pages (40 articles). CQ = Clinical Queries, PIC(O) = population, interventions, comparison, (outcomes).

The sensitivity and the positive predictive value (PPV) were also highly variable within each search strategy (Figure 3). When the full outputs were screened for relevant studies, about 85% were detected by PIC queries and 69% by PICO queries (Figure 3A). Overall sensitivity remained comparable when we used Clinical Queries filters (with or without limits), although the Clinical Queries narrow filter was associated with slightly lower overall sensitivities (Table 1A). In contrast, the use of the AIM limit systematically lowered sensitivity to about 15% (Figure 3A). The overall sensitivity of searches based on related articles was also extremely variable, with a median of about 60%.

When the screening of relevant articles was limited to the first 2 pages (ie, 40 articles), sensitivity dropped to 10% for unfiltered searches and PPV decreased to 2.5%–10% (Figure 3B). In contrast, all search strategies that used the Clinical Queries narrow filter (S4, S5, S10, and S11 in Table 2) showed significantly higher sensitivities, overall around 50% (*P *< .001 when compared with their corresponding unfiltered queries) and higher PPV values, between 20% and 30% (*P *< .001) within the first 2 pages of the output. When looking at the 30 questions individually, adding a Clinical Queries narrow filter to a query increased the sensitivity of the search for 21 questions (70%), kept it stable for 6 (20%), and decreased it for 3 (10%), whereas PPV increased in 27 questions (90%) (Table 1B). Moreover, adding a Clinical Queries narrow filter to a PIC query reduced the risk of finding no relevant articles in the first 2 pages from 11 questions (37%) to 4 questions (13%). The broad Clinical Queries filter did not improve search performance; nor did searches using related articles and the additional use of limits. Overall, PICO queries had slightly higher performances than PIC queries, although the differences were not statistically significant.

**Figure 4:**
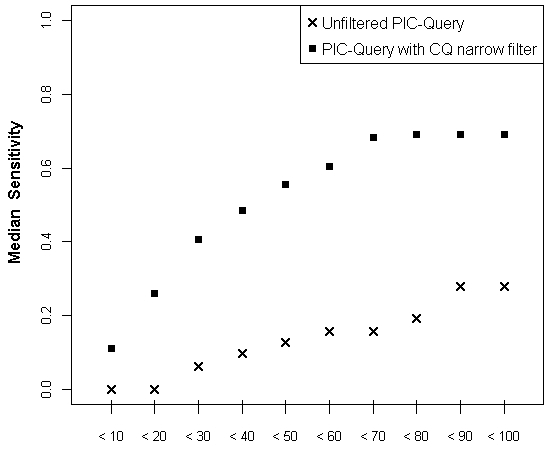
Evolution of median sensitivity of the search according to the number of studies screened in the output for the unfiltered population, interventions, comparison (PIC) query (S1) and the PIC query filtered by the Clinical Queries (CQ) narrow filter (S4).

The exploration of search performance according to the number of articles screened in the output showed that sensitivity rose gradually up to about 100 items in unfiltered searches (Figure 4). In contrast, the sensitivity of searches using the Clinical Queries narrow filter rose much more steeply, peaking earlier at 50 to 60 articles screened. Finally, we found no significant association between search performance and characteristics of the reviews, namely the absolute number of relevant articles included, the number of PICO search terms required to summarize its question, or the presence of mortality as an outcome (data not shown).

**Table 2:** Searches strategies: description, number of hits, and performances over the first 2 pages of PubMed output.

Strategy No.	Search strategy			No. of hits in output		Performances for an output of 2 pages (maximum 40 items)				
						Sensitivity (%)		PPV^b ^(%)		NNR^c^
	Query	Clinical Queries	Limits	Median	IQR^a^	Median	IQR	Median	IQR	Median
S1	PIC^d^	NA^e^	NA	173	79–322	9.8	0.0–29.4	2.5	0.0–10.0	40
S2	PIC	Therapy, broad	NA	126	66–276	14.6	0.0–30.0	5.0	0.0–10.0	20
S3	PIC	Therapy, broad	English, human	97	59–229	17.6	0.0–33.3	5.0	0.0–12.5	20
S4	PIC	Therapy, narrow	NA	33	17–67	48.5	23.5–80.0	21.3	12.5–35.0	5
S5	PIC	Therapy, narrow	English, human	31	14–67	52.8	23.5–80.0	23.8	12.5–36.4	4
S6	PICO^d^	NA	NA	91	36–179	17.9	4.3–60.0	6.3	2.5–15.0	16
S7	PICO	Therapy, broad	NA	75	33–165	26.1	9.1–75.0	8.8	5.0–20.0	11
S8	PICO	Therapy, broad	English, human	62	28–138	29.6	9.1–75.0	11.3	5.0–20.8	9
S9	PICO	Therapy, broad	English, human, AIM^f^	11	5–24	15.5	11.1–40.0	20.0	14.3–33.3	5
S10	PICO	Therapy, narrow	NA	22	13–51	54.7	27.3–78.6	32.1	14.3–50.0	3
S11	PICO	Therapy, narrow	English, human	20	12–50	54.7	27.3–78.6	32.8	15.0–50.0	3
S12	PICO	Therapy, narrow	English, human, AIM	5	3–13	15.5	10.5–33.3	50.0	23.1–56.2	2
S13	Related #1^g^	NA	NA	350	204–599	39.7	20.0–50.0	10.0	5.0–15.0	10
S14	Related #2^g^	NA	NA	340	138–484	37.9	18.4–62.5	10.0	5.0–17.5	10
S15	Related #3^g^	NA	NA	305	167–558	37.5	18.5–50.0	7.5	5.0–17.5	13

^a ^Interquartile range.

^b ^Positive predictive value (ie, precision).

^c ^Number of items needed to read to find a relevant article in the screened output; equal to 1/PPV of the search.

^d ^Population, interventions, comparison, (outcomes).

^e ^Not applied.

^f ^Abridged Index Medicus (set of 119 core clinical journals [28]).

^g ^From the output of search S11, related articles were searched for the 3 articles whose title was closest to the PICO query.

## Discussion

The performance of the search strategies tested was highly variable according to the clinical question. None of the 15 strategies showed a consistently high sensitivity in retrieving relevant articles identified by systematic reviews. Even the best strategies had a sensitivity of about 50%, with a range from 0% to 100% across the 30 questions. Therefore, clinicians who perform a specific PubMed search cannot foresee to what extent their search will be successful.

Nevertheless, on average, some search strategies were more successful than others. Unfiltered searches based on PIC or PICO queries, as well as those using the broad Clinical Queries filter, produced large outputs and retrieved few relevant articles within the first 2 pages. In contrast, searches that used the Clinical Queries narrow filter had a significantly higher sensitivity (around 50%–55%) and a higher PPV (around 20%–30%). These improved performances were observed for most clinical questions. Additional use of limits had only a marginal effect, except for the limit to core medical journals, which actually reduced sensitivity. Finally, searches based on related articles retrieved about a third of relevant studies in the first 2 pages. However, their PPV remained low, despite the ranking of citations by content similarity, because they also retrieved hundreds of nonrelevant articles. This may be because related articles will also retrieve studies that are not randomized trials, in contrast with Clinical Queries therapeutic filters.

Clinicians who use PubMed at the point of care favor short queries of 2–3 terms, without any limits or filters, and generally screen only the first 2 pages of the output (ie, 20–40 items) [19,31]. Our results clearly show that such strategies will miss many relevant articles. Although these strategies may reach high overall sensitivities, relevant citations will be scattered over many pages that will not be screened in real life. To increase the density of relevant articles in the output (ie, the PPV), we recommend the use of the PIC(O) framework. Such queries including 5–8 terms kept a satisfactory overall sensitivity of about 85% (Figure 3A). However, when only the first 2 pages of the output were scanned, which is what usually occurs in clinical practice [21], this sensitivity dropped to about 10%, with an even lower PPV of 2.5% (corresponding to a median of 1 relevant article per screened output).

To improve search performance within readable outputs, an important finding is the usefulness of the Clinical Queries narrow therapy filter in identifying relevant studies in the first 2 pages of PubMed output. Clinical Queries filters were designed by Haynes et al [27] based on a hand search of 161 clinical journals that identified methodologically sound randomized controlled trials. When applied to the whole Medline database, the Clinical Queries broad filter retained 99% of hand-selected studies, while the narrow filter retained 93% [27]. Hoogendam et al found similar results [31]. Our study confirmed the high sensitivity of the Clinical Queries filters when applied to specific clinical questions. A surprising finding was the increased sensitivity of the narrow filter compared with the broad filter when we screened only a realistic portion of the search output; this was because the narrow filter concentrated relevant articles on the first pages of the output. In other words, sensitivity, assessed on 2 pages of output, was better as PPV improved.

Based on our results we recommend that clinicians at the point of care start their PubMed searches by formulating a PIC query combined with a narrow filter, and then adapt it according to the output size and relevancy of the first items screened. If this strategy retrieves too many irrelevant citations, search terms describing the outcome (full PICO query) and further search limits can be added. In the opposite case, filters can be deactivated and sensitivity can be gained by adding search terms or by using the related articles link from the first relevant studies found. Finally, a general recommendation would be to screen beyond the first page of the output, as even the most efficient strategies require at least 50 to 60 items to approach their maximum sensitivity (Figure 4).

### Study Limitations and Strengths

The main limitation of our study is that we assessed search strategies based solely on their retrieval performance. The searches were not performed by clinicians at the point of care, so we did not capture the iterative process of searching PubMed, based on trial and error [43,44], and could not assess whether relevant articles would be identified in practice. Furthermore, we did not examine the impact of search strategies on medical decisions and patients’ outcomes. However, performing searches that retrieve relevant articles is a prerequisite for their further use in clinical practice. Moreover, by using predefined search procedures, we were able to isolate and compare the effect of several components of search strategies in relation to a specific clinical question. As we focused on retrieval, we considered each relevant article to be equally important, although this may not be the case. In particular, we did not examine selection bias attributable to suboptimal identification of relevant articles. Finally, our results apply only to therapeutic interventions, assessed by clinical trials, and cannot be generalized to questions on prevention or diagnosis [32]. For the latter, similar studies could be conducted using alternative Clinical Queries filters designed to improve the retrieval of high-quality studies on diagnosis or prognosis [26].

 Among its strengths, our design allowed an unbiased comparison of strategies, by controlling important sources of variation such as clinicians’ searching skills or previous knowledge [29,30]. This exploration was broader and more systematic than in previous studies. Moreover, the tested search strategies relied on search components that are easily applicable in practice, as they are directly implemented in PubMed. Finally, we used reputable systematic reviews to identify relevant studies on specific clinical topics.

### Conclusion and Future Prospects

Countless PubMed searches are performed daily by clinicians, but the effectiveness of this activity is poorly understood [20]. We have shown that a well-formulated PIC query used with the Clinical Queries narrow filter was most likely to retrieve relevant clinical trials within readable outputs. These results can help clinicians build more effective strategies to answer their questions at the point of care, and thus reduce the gap between evidence from clinical trials and its actual implementation in practice. Further research should focus on the performance and clinical usefulness of selected strategies when they are performed in real practice [45]. Meanwhile, PubMed remains a perfectible tool. Areas of improvement include the development of content filters for specific clinical disciplines [46], or the implementation of new search interfaces that help clinicians formulate effective queries [24] and conduct parallel searches combining methodological and content filters at will.

## Multimedia Appendix 1

Detailed description of the 30 Cochrane systematic reviews on therapeutic interventions, used to identify relevant studies on 30 clinical questions.

## Multimedia Appendix 2

Two examples of search term extraction forms for one complex (review #1) and one simpler (review #7) clinical questions.

## Multimedia Appendix 3

Detailed search terms for 30 clinical questions, extracted from the abstract of the corresponding Cochrane systematic review according to the PICO framework.

## Abbreviations

AIM: Abridged Index Medicus
COPD: chronic obstructive pulmonary disease
IQR: interquartile range
MeSH: Medical Subject Headings
PIC(O): population, interventions, comparison, (outcomes)
PPV: positive predictive value
