# Supplementary material for: Sensitivity and Predictive Value of 15 PubMed Search Strategies to Answer Clinical Questions Rated Against Full Systematic Reviews
Source: J Med Internet Res. 2012 Jun 12;14(3):e85. doi: 10.2196/jmir.2021 (PMC3414859; doi:10.2196/jmir.2021)
Supplement: Supplementary file 1 [file jmir_v14i3e85_app1.pdf]

Multimedia Appendix 1. Detailed description of the 30 Cochrane Systematic Reviews on therapeutic interventions, used to identify relevant studies on 30 clinical questions.

	PMID	Author	itle	Cochrane Group	Issue (2010)	Review status (as of February 2010)	Year(Month) content uptodate	N studies included in review	N (%) retrievable in Pubmed
**1**	12076427	Tudur	Carbamazepine versus phenytoin monotherapy for epilepsy	Epilepsy	2	New search: no change	2010 (1)	10	8 (80)
**2**	20166082	Yang	Chest physiotherapy for pneumonia in adults	Acute Respiratory Infections	2	New review	2009 (8)	6	5 (83)
**3**	17443601	Vergara	Epinephrine injection versus epinephrine injection and a second endoscopic method in high risk bleeding ulcers	Upper Gastrointestinal and Pancreatic	2	New search: no change	2009 (12)	18	15 (83)
**4**	20166076	Fluri	Extracranial-intracranial arterial bypass surgery for occlusive carotid artery disease	Stroke	2	New review	2009 (10)	21	18 (86)
**5**	16625634	Lasserson	Fluticasone versus 'extrafine' HFA-beclomethasone dipropionate for chronic asthma in adults and children	Airways	2	New search: no change	2010 (1)	9	8 (89)
**6**	20166073	Thomas	Influenza vaccination for healthcare workers who work with the elderly	Acute Respiratory Infections	2	New search: conclusions changed	2009 (9)	5	5 (100)
**7**	20166060	Poole	Mucolytic agents for chronic bronchitis or chronic obstructive pulmonary disease	Airways	2	New search: no change	2008 (10)	29	18 (62)

	PMID	Author	Title	Cochrane Group	Issue (2010)	Review status (as of February 2010)	Year(Month) content uptodate	N studies included in review	N (%) retrievable in Pubmed
**8**	20166059	Jefferson	Neuraminidase inhibitors for preventing and treating influenza in healthy adults	Acute Respiratory Infections	2	New search: conclusions changed	2009 (8)	20	20 (100)
**9**	20091650	van den Aardweg	Adenoidectomy for otitis media in children	Ear, Nose and Throat	1	New review	2009 (10)	14	14 (100)
**10**	20091548	O'Meara	Antibiotics and antiseptics for venous leg ulcers	Wounds	1	New search: no change	2009 (11)	25	13 (52)
**11**	20091544	Abraham	Antithyroid drug regimen for treating Graves' hyperthyroidism	Metabolic and Endocrine Disorders	1	New search: no change	2009 (4)	27	23 (85)
**12**	17943870	Jones	Artesunate versus quinine for treating severe malaria	Infectious Diseases	1	New search:no change	2007 (6)	6	4 (67)
**13**	17443530	Herkner	Bed rest for acute uncomplicated myocardial infarction	Heart	1	New search: no change	2009 (10)	17	11 (65)
**14**	20091630	Simon	Benzodiazepines for the relief of breathlessness in advanced malignant and non-malignant diseases in adults	Pain, Palliative and Supportive Care	1	New review	2009 (9)	7	4 (57)
**15**	20091622	Chen	Blood pressure lowering efficacy of beta-blockers as second-line therapy for primary hypertension	Hypertension	1	New review	2009 (11)	20	11 (55)
**16**	20091662	Heran	Blood pressure lowering efficacy of potassium-sparing diuretics (that block the epithelial sodium channel) for primary hypertension	Hypertension	1	New review	2009 (10)	8	7 (88)

/

	PMID	Author	Title	Cochrane Group	Issue (2010)	Review status (as of February 2010)	Year(Month) content uptodate	N studies included in review	N (%) retrievable in Pubmed
**17**	20091514	Welsh	Caffeine for asthma	Airways	1	New search: no change	2009 (8)	7	7 (100)
**18**	20091571	Misso	Continuous subcutaneous insulin infusion (CSII) versus multiple insulin injections for type 1 diabetes mellitus	Metabolic and Endocrine	1	New review	2009 (7)	34	27 (79)
**19**	20091657	Robert	Effect of cyclosporine on blood pressure	Hypertension	1	New review	2009 (6)	17	17 (100)
**20**	20091534	Al-Omran	Enteral versus parenteral nutrition for acute pancreatitis	Upper Gastrointestinal and Pancreatic	1	New search: conclusions changed	2009 (10)	8	8 (100)
**21**	20091596	Choi	Exercises for prevention of recurrences of low-back pain	Back	1	New review	2009 (7)	13	13 (100)
**22**	20091618	Taylor Rod	Home-based versus centre-based cardiac rehabilitation	Heart	1	New review	2008 (7)	22	17 (77)
**23**	20091617	Powell	Immediate-release versus controlled-release carbamazepine in the treatment of epilepsy	Epilepsy	1	New review	2009 (11)	10	10 (100)
**24**	17054257	Sreedharan	Proton pump inhibitor treatment initiated prior to endoscopic diagnosis in upper gastrointestinal bleeding	Upper Gastrointestinal and Pancreatic	1	New search: no change	2009 (8)	6	3 (50)
**25**	20091560	Marshall	Rectal 5-aminosalicylic acid for induction of remission in ulcerative colitis	Inflammatory Bowel Disease and Functional Bowel	1	New review	2008 (12)	38	33 (87)
**26**	18646149	Cates	Regular treatment with salmeterol for chronic asthma: serious adverse events	Airways	1	New search: no change	2009 (8)	49	30 (61)

/

	PMID	Author	Title	Cochrane Group	Issue (2010)	Review status (as of February 2010)	Year(Month) content uptodate	N studies included in review	N (%) retrievable in Pubmed
**27**	17943842	Davies	Rifabutin for treating pulmonary tuberculosis	Infectious Diseases	1	New search: no change	2007 (7)	5	4(80)
**28**	20091591	Billio	Serotonin receptor antagonists for highly emetogenic chemotherapy in adults	Pain, Palliative and Supportive Care	1	New review	2009 (3)	16	15(94)
**29**	16855986	van Pinxteren	Short-term treatment with proton pump inhibitors, H2-receptor antagonists and prokinetics for gastro-oesophageal reflux disease-like symptoms and endoscopy negative reflux disease	Upper Gastrointestinal and Pancreatic	1	New search: no change	2009 (10)	45	38 (80)
**30**	20091539	Rutjes	Therapeutic ultrasound for osteoarthritis of the knee or hip	Musculoskeletal	1	New search: conclusions changed	2009 (7)	5	5 (100)

/
