# Supplementary material for: Sensitivity and Predictive Value of 15 PubMed Search Strategies to Answer Clinical Questions Rated Against Full Systematic Reviews
Source: J Med Internet Res. 2012 Jun 12;14(3):e85. doi: 10.2196/jmir.2021 (PMC3414859; doi:10.2196/jmir.2021)
Supplement: Supplementary file 2 [file jmir_v14i3e85_app2.pdf]

Multimedia Appendix 2. Two examples of search terms extraction forms for one complex (review #1) and one simpler (review # 7) clinical questions.

**Review number**		**1**
**Title**		**Carbamazepine versus phenytoin monotherapy for epilepsy**
PMID		12076427
Link		http://www.mrw.interscience.wiley.com/cochrane/clsysrev/articles/CD001911/frame.html
ABSTRACT: Objective		To review the best evidence comparing carbamazepine and phenytoin when used as monotherapy in people with partial onset seizures, or generalized onset tonic-clonic seizures with or without other generalized seizure types.
ABSTRACT: Selection Criteria		Randomized controlled trials in children or adults with partial onset seizures or generalized onset tonic-clonic seizures. Trials must have included a comparison of carbamazepine monotherapy with phenytoin monotherapy.
METHODS: Types of outcomes		(1) Time to withdrawal of allocated treatment (retention time) was chosen as the primary outcome. This is a combined outcome reflecting both efficacy and tolerability as treatment may be withdrawn due to continued seizures, side effects, non-compliance or if additional add-on treatment was initiated (ie allocated treatment had failed). This is an outcome to which the patient makes a contribution, and is the primary outcome measure recommended by the Commission on Antiepileptic Drugs of the International League Against Epilepsy<br>(2) Time to achieve 12 month remission (seizure free period).<br>(3) Time to achieve six month remission.<br>(4) Time to first seizure post randomization.
**P**	Epilepsy<br>Partial onset seizures<br>Generalized onset tonic-clonic seizures<br>with or without other generalized seizure types.<br>children or adult	à (epilepsy) OR (partial onset seizures) OR (generalized onset tonic-clonic seizures)
**I**	Carbamazepine monotherapy	à (carbamazepine monotherapy)
**C**	Phenytoin monotherapy	à (phenytoin monotherapy)
**O**	(1) withdrawal of allocated treatment<br>(2) 12 month remission<br>(3) six month remission<br>(4) first seizure post randomization.	à (withdrawal treatment) OR (remission) OR (seizure)
**Retained PICO Query**		**((Epilepsy) OR (partial onset seizures) OR (generalized onset tonic-clonic seizures)) AND (carbamazepine monotherapy) AND (phenytoin monotherapy) AND ((withdrawal treatment) OR (remission) OR (seizure))**

PubMed translation of search terms (automating mapping)

**Epilepsy**	epilepsy[MeSH Terms] OR "epilepsy"[All Fields]
**seizures**	seizures[MeSH Terms] OR "seizures"[All Fields]
**tonic-clonic seizures**	seizures[MeSH Terms] OR "seizures"[All Fields] OR ("tonic"[All Fields] AND "clonic"[All Fields] AND "seizures"[All Fields]) OR "tonic clonic seizures"[All Fields]
**carbamazepine**	carbamazepine[MeSH Terms] OR "carbamazepine"[All Fields]
**phenytoin**	phenytoin[MeSH Terms] OR "phenytoin"[All Fields]
**treatment**	therapy[Subheading] OR "therapy"[All Fields] OR "treatment"[All Fields] OR "therapeutics"[MeSH Terms] OR "therapeutics"[All Fields]
**seizure**	seizures[MeSH Terms] OR "seizures"[All Fields] OR "seizure"[All Fields]

	**7**

Review number	
**Title**	**Mucolytic agents for chronic bronchitis or chronic obstructive pulmonary disease**
PMID	20166060
Link	http://www.mrw.interscience.wiley.com/cochrane/clsysrev/articles/CD001287/frame.html
ABSTRACT: Objective	To assess the effects of oral mucolytics in adults with stable chronic bronchitis or COPD.
ABSTRACT: Selection Criteria	Randomised trials that compared oral mucolytic therapy with placebo for at least two months in adults with chronic bronchitis or COPD. We excluded studies of people with asthma and cystic fibrosis.
METHODS: Types of outcomes	Primary outcomes<br>1. The number of acute exacerbations, defined as an increase in cough and in the volume and/or purulence of sputum. This was also represented by the number of patients with no exacerbations in the study period.<br>2. The number of days of disability variously defined as days in bed, days off work or days where the subject was unable to undertake normal activities. Days on antibiotics were also assessed.
**P** Chronic bronchitis or chronic obstructive pulmonary disease<br>Adults<br>Stable chronic bronchitis or COPD<br>Excluded studies of people with asthma and cystic fibrosis	à ((chronic bronchitis) OR COPD)
**I** Mucolytic agents<br>Oral mucolytic therapy<br>At least two months	à (mucolytics)
**C** Placebo	à placebo
**O** Number of acute exacerbations defined as an increase in cough and in the volume and/or purulence of sputum<br>Number of days of disability	à exacerbations
**Retained PICO Query**	**((chronic bronchitis) OR COPD) AND (mucolytics) AND placebo AND exacerbations**

PubMed translation of search terms (automating mapping)

**chronic bronchitis**	bronchitis, chronic[MeSH Terms] OR ("bronchitis"[All Fields] AND "chronic"[All Fields]) OR "chronic bronchitis"[All Fields] OR ("chronic"[All Fields] AND "bronchitis"[All Fields])
**COPD**	pulmonary disease, chronic obstructive[MeSH Terms] OR ("pulmonary"[All Fields] AND "disease"[All Fields] AND "chronic"[All Fields] AND "obstructive"[All Fields]) OR "chronic obstructive pulmonary disease"[All Fields] OR "copd"[All Fields]
**mucolytics**	expectorants[MeSH Terms] OR "expectorants"[All Fields] OR "mucolytics"[All Fields] OR "expectorants"[Pharmacological Action]
**placebo**	placebos[MeSH Terms] OR "placebos"[All Fields] OR "placebo"[All Fields]
