# Supplementary material for: Sensitivity and Predictive Value of 15 PubMed Search Strategies to Answer Clinical Questions Rated Against Full Systematic Reviews
Source: J Med Internet Res. 2012 Jun 12;14(3):e85. doi: 10.2196/jmir.2021 (PMC3414859; doi:10.2196/jmir.2021)
Supplement: Supplementary file 3 [file jmir_v14i3e85_app3.pdf]

Multimedia Appendix 3. Detailed search terms for 30 clinical questions, extracted from the abstract of the corresponding Cochrane Systematic Review according to the PICO-framework.

	Population	Intervention	Control	Outcome
1	epilepsy OR<br>(partial onset seizures) OR<br>(generalized onset tonic-clonic seizures)	carbamazepine monotherapy	phenytoin monotherapy	(withdrawal treatment) OR<br>remission OR<br>seizure
2	pneumonia AND adults	physiotherapy		cure OR<br>mortality OR<br>(adverse events) OR<br>duration
3	(peptic ulcer) AND<br>(bleeding OR haemorrhage)	epinephrine injection	endoscopic treatment	rebleeding OR<br>surgery OR<br>complications OR<br>mortality
4	carotid AND (occlusion OR symptomatic)	extracranial-intracranial bypass surgery		ischemic OR<br>stroke OR<br>haemodynamic OR<br>dependency
5	asthma	fluticasone	beclomethasone AND<br>(extrafine OR HFA)	(lung function) OR<br>spirometry OR<br>exacerbation
6	((health care workers) OR<br>(long-term care facilities) OR<br>(nursing homes))<br>AND (elderly OR residents)	influenza vaccination		incidence OR<br>complication OR<br>mortality
7	(chronic bronchitis) OR COPD	mucolytics	placebo	exacerbations

/

	Population	Intervention	Control	Outcome
**8**	influenza AND adults	(neuraminidase inhibitors) OR<br>oseltamivir OR<br>zanamivir	placebo	treatment OR<br>prevention OR<br>transmission OR<br>complications OR<br>(adverse events)
**9**	(otitis media) AND children	adenoidectomy		effusion OR time
**10**	venous leg ulcer	antibiotic OR antiseptic		healing OR<br>surface OR<br>size
**11**	Graves' hyperthyroidism	(antithyroid drug therapy) OR<br>carbimazole OR<br>propylthiouracil OR<br>methimazole	dose OR<br>regimen OR<br>duration	relapse OR<br>hypothyroidism OR<br>mortality
**12**	severe malaria	artesunate	quinine	mortality
**13**	uncomplicated myocardial infarction	(bed rest) OR (early mobilisation)		mortality OR reinfarction
**14**	advanced OR chronic	benzodiazepine		breathlessness OR<br>dyspnoea
**15**	hypertension AND adult	beta-blocker AND combination	antihypertensive AND<br>(alone OR monotherapy)	reduction OR<br>(heart rate) OR<br>(adverse effects)
**16**	hypertension	amiloride OR triamterene	placebo OR antihypertensive	(blood pressure lowering)<br>OR<br>(adverse effects)
**17**	asthma AND adults	caffeine OR coffee	placebo OR decaffeinated	(lung function) OR<br>(exhaled nitric oxide)
**18**	type 1 diabetes	continuous subcutaneous insulin infusion	multiple insulin injection	(blood glucose) OR<br>HbA1c OR<br>(hypoglycaemic episodes)<br>OR (quality of life)
**19**		cyclosporin	placebo	blood pressure

/

	Population	Intervention	Control	Outcome
**20**	acute pancreatitis	enteral nutrition	parenteral nutrition	mortality OR<br>(length of stay) OR<br>SIRS OR<br>MOF OR<br>complications
**21**	low-back pain	exercise OR (physical activity)		recurrence
**22**	(myocardial infarction) OR<br>(coronary disease) OR<br>(heart failure)	home based rehabilitation	centre OR hospital rehabilitation	mortality OR<br>(quality of life) OR<br>(risk factors)
**23**	epilepsy	carbamazepine	controlled release	seizure OR (adverse events)
**24**	upper gastrointestinal bleeding	(proton pump inhibitor) AND<br>(before endoscopy)		mortality OR<br>rebleeding OR<br>surgery
**25**	ulcerative colitis	5-ASA AND<br>(rectal OR suppository OR enema)		remission
**26**	asthma	salmeterol		(adverse events) OR mortality
**27**	tuberculosis	rifabutin	rifampicin	culture OR conversion
**28**	chemotherapy AND adult AND cancer	(serotonin OR 5-HT3) AND antagonists		nausea OR vomiting
**29**	(gastro-oesophageal reflux disease) OR heartburn	(proton pump inhibitor) OR<br>(H2-receptor antagonist) OR<br>prokinetic		remission OR<br>relief OR<br>(quality of life)
**30**	osteoarthritis AND (knee OR hip)	therapeutic ultrasound		pain OR function
